# MgFe‐LDH Nanoparticles: A Promising Leukemia Inhibitory Factor Replacement for Self‐Renewal and Pluripotency Maintenance in Cultured Mouse Embryonic Stem Cells

**DOI:** 10.1002/advs.202003535

**Published:** 2021-02-25

**Authors:** Xiaolie He, Yanjing Zhu, Li Yang, Zhaojie Wang, Zekun Wang, Jianhao Feng, Xuejun Wen, Liming Cheng, Rongrong Zhu

**Affiliations:** ^1^ Key Laboratory of Spine and Spinal Cord Injury Repair and Regeneration of Ministry of Education Orthopaedic Department of Tongji Hospital School of Life Science and Technology Tongji University 389 Xincun Road Shanghai 200065 P. R. China; ^2^ Department of Chemical and Life Science Engineering School of Engineering Virginia Commonwealth University Richmond VA 23284 USA

**Keywords:** combined transcriptomic and proteomic analysis, embryonic stem cells, LIFR/JAK/STAT3, MgFe‐LDH nanoparticles, pluripotency

## Abstract

Leukemia inhibitory factor (LIF), an indispensable bioactive protein that sustains self‐renewal and pluripotency in stem cells, is vital for mouse embryonic stem cell (mESC) culture. Extensive research is conducted on reliable alternatives for LIF as its clinical application in stable culture and large‐scale expansion of ESCs is limited by its instability and high cost. However, few studies have sought to replace LIF with nanoparticles to provide a xeno‐free culture condition. MgAl‐LDH (layered double hydroxide) nanoparticles can partially replace LIF in maintaining pluripotency of mESCs; however, the requirement and tolerance for aluminum ions in mice are far lesser than those of iron ions. Hence, MgFe‐LDH nanoparticles are selected for this study. MgFe‐LDH is superior to MgAl‐LDH in maintaining self‐renewal and pluripotency of mESCs, in the absence of LIF and mouse embryonic fibroblast. Furthermore, combined transcriptomic and proteomic analysis confirms that MgFe‐LDH can activate the LIF receptor (LIFR)/phosphatidylinositol 3‐kinase (PI3K)/protein kinase B(AKT), LIFR/JAK/janus kinase (JAK)/signal transducer and activator of transcription 3(STAT3), and phospho‐signal transducer and activator of transcription 3(p‐STAT3)/ten‐eleven translocation (TET) signaling pathways, while the extra Fe^2+^ provided by MgFe‐LDH would also enhance TET1/2 abundance thus affecting the TET1/2 regulated pluripotency related marker expression and TET1/2 meditated DNA demethylation. These results suggest that MgFe‐LDH nanoparticles can thus be used as an affordable and efficient replacement for LIF in mESC cultivation.

## Graphical abstract

The role of MgFe‐LDH (layered double hydroxide) nanoparticles as promising leukemia inhibitory factor (LIF) replacement in maintaining mouse embryonic stem cells (mESCs) self‐renewal and pluripotency as compared with MgAl‐LDH was explored, MgFe‐LDH is superior to MgAl‐LDH. Combined transcriptomic and proteomic analysis reveals the underling mechanism. MgFe‐LDH favors economical and efficient replacement for LIF in large‐scale expansion of mESCs.

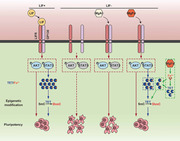

## 1 Introduction

Embryonic stem cells (ESCs) are characterized by self‐renewal and pluripotency. They have been successfully differentiated into several cell types—including nerve cells, retinal pigmented epithelium, cardiomyocytes, islet cells, and liver cells—that are used in the therapy of spinal cord injury,^[^ ^1^, ^2^ ^]^ ophthalmic diseases,^[^ ^3^, ^4^ ^]^ cardiovascular disease,^[^ ^5^ ^]^ diabetes, and liver injury,^[^ ^6^, ^7^, ^8^ ^]^ respectively. The traditional cell culture method employed to culture mouse embryonic stem cells (mESCs) requires the participation of leukemia inhibitory factor (LIF);^[^ ^9^ ^]^ withdrawing LIF from the culture medium would result in the spontaneous differentiation of mESCs. As a member of the interleukin‐6 family, LIF can bind to LIF receptor (LIFR) and further function via the janus kinase (JAK)/signal transducer and activator of transcription 3 (STAT3), phosphatidylinositol 3‐kinase (PI3K)/protein kinase B (AKT), and SHP2/mitogen‐activated protein kinases (MAPK) signaling pathways to sustain mESC pluripotency.^[^ ^10^ ^]^ Nevertheless, the high cost and instability of recombinant LIF protein limits its clinical application in the large‐scale cultivation of pluripotent stem cells. Thus, there is extensive research currently underway on new approaches that focus on improving the existing culture method.

It has been proven that biological factors (growth factors and small molecule drugs) as well as physical cues (topography, stiffness, and wettability) are integral to ESC pluripotency and differentiation.^[^ ^11^, ^12^, ^13^, ^14^, ^15^ ^]^ In recent times, biomaterials are being widely employed in improving the existing cell culture techniques. For example, reduced graphene oxide substrates were used to maintain both human and mouse ESC pluripotency in the absence of feeders in vitro by regulating the E‐cadherin‐mediated cell–cell interaction and Wnt signaling pathway.^[^ ^16^ ^]^ A protein‐based polymer substrate mimicking the capability of natural fibronectin in retaining stemness was developed for the long‐term culture of hPSCs.^[^ ^17^ ^]^ Gold nanoparticle layers with nano‐ and sub‐microscale surface roughnesses markedly supported mESCs pluripotency while microscale surface roughness resulted in unidirectional differentiation, which emphasized the role of topographies on stemness maintenance.^[^ ^18^ ^]^ Although nanoparticles have received a lot of attention in regulating the pluripotency of ESCs, previous studies have primarily focused on developing new substrates to replace mouse embryonic fibroblast (MEF) in ESC culture, and few sought to replace LIF and provide a xeno‐free culture environment. In this study, we aimed to optimize a chemically well‐defined biomaterial for mESC culture.

Layered double hydroxide (LDH) nanoparticles are a type of anionic hydrotalcite‐like clay materials, and usually defined as [M^2+^_1−_ *_x_*M^3+^ *_x_*(OH)_2_]*^x^* ^+^[A^p−^] *_x_*_/_ *_p_*·mH_2_O, in which M stands for metal ions. Different combinations of metal ions in LDH would affect the physicochemical properties of LDH, in turn influencing the fate of cells cultured with LDH nanoparticles.^[^ ^19^, ^20^ ^]^ LDH has been widely used in biomedical research and applications, due to its superior biocompatibility and low cytotoxicity.^[^ ^21^, ^22^, ^23^ ^]^ One of our previous studies revealed that MgAl‐LDH can partly replace LIF thus sustaining the pluripotency of mESCs by activating the PI3K/AKT signaling pathway.^[^ ^24^, ^25^ ^]^ Since MgAl‐LDH did not effectively activate JAK/STAT3, it has not yet fully replaced LIF in mESC culture. Apart from the lower requirement and tolerance for aluminum ions than iron ions in mice, it has been reported that iron ions can also affect the self‐renewal of ESCs.^[^ ^26^, ^27^, ^28^, ^29^ ^]^ Hence, we proposed to replace Al ions with Fe ions for LDH modification.

This study aimed to synthesize MgFe‐LDH nanoparticles and analyze the role of MgFe‐LDH nanoparticles in regulating the self‐renewal and differentiation potential of mESCs as compared with MgAl‐LDH and LIF. Specifically, the efficiency of MgFe‐LDH nanoparticles in supporting mESC self‐renewal was achieved in LIF and MEF‐free conditions. In addition, combined transcriptomic and proteomic analysis was employed to reveal the molecular mechanisms underlying the effects of MgFe‐LDH nanoparticles compared with that of either MgAl‐LDH or LIF. MgFe‐LDH nanoparticles not only provide a route for easier and economical mESC culture (based on our calculations, LIF is 3.78 × 10^4^ times as high as MgFe‐LDH in price, when we use 20 µg mL^−1^ MgFe‐LDH) but also offer improved quality and a promising replacement for LIF in maintaining the pluripotency of cultured mESCs, in a chemically defined manner.

## 2 Results

### 2.1 Synthesis and Characterization of MgFe‐LDH and MgAl‐LDH Nanoparticles

The internal lattice structure of the nanoparticles was characterized using transmission electron microscope (TEM). The resultant TEM images indicated that both the MgFe‐LDH and MgAl‐LDH nanoparticles are layered hexagonally (Figure S1A, Supporting Information). The surface morphology was further observed via scanning electron microscope (SEM) and was consistent with the TEM results. Both the MgFe‐LDH and MgAl‐LDH nanoparticles were observed to be hexagonal, with a specific lamellar morphology (Figure S1B, Supporting Information). Moreover, the chemical composition and crystal phase of the LDH were assessed by X‐ray diffraction (XRD) (Figure S1C, Supporting Information). MgFe‐LDH as well as MgAl‐LDH exhibited sharp, symmetrical, and narrow characteristic peaks of (003), (006), and (009) in the XRD spectrum, demonstrating the successful construction of nanomaterials with a completely layered structure. Based on the vibrational patterns of the interacting infrared light and molecules, the anion types as well as bonding types were measured via Fourier‐transform infrared (FTIR) spectroscopy (Figure S1D, Supporting Information). For MgFe‐LDH and MgAl‐LDH, the stretching vibration peaks of O‐H occurred at 3482.12 and 1384.41 cm^−1^, respectively, while for NO_3_^−^ they were at 3456.14 and 1384.37 cm^−1^, respectively. Thus, there was no significant difference between the peaks. The average diameters of the MgFe‐LDH and MgAl‐LDH nanoparticles were 107.9 ± 4.7 and 111.7 ± 8.2 nm, respectively (Figure S1E, Supporting Information); there was no significant difference in the diameters of the nanoparticles synthesized by the same method and it has been previously proven that LDH with different particle sizes would determine cell pluripotency or cell fate.^[^ ^30^ ^]^ Additionally, the surface charges of the nanoparticles were detected (Figure S1F, Supporting Information), wherein the average zeta potentials of MgFe‐LDH and MgAl‐LDH were 17.9 and 30.7 mV, respectively; this would facilitate the adhesion of nanoparticles to negatively charged cell membranes.

### 2.2 Biocompatibility Comparison of MgFe‐LDH and MgAl‐LDH Nanoparticles in mESCs

The survival rate of mESCs treated with nanoparticles for 24 and 48 h was assessed via a cell counting kit‐8 (CCK‐8) assay. No significant differences in cell viability were observed between the control and LDH at different doses (Figure S2A, Supporting Information). Given that LDH can bind to the negatively charged cell membrane of mESCs, lactic dehydrogenase release from the cytoplasm was detected as an indication of comprised cell membrane integrity following nanoparticle treatment (Figure S2B, Supporting Information). Compared with the LIF+ group, none of the LDHs at different doses exhibited any significant change in lactic dehydrogenase release from mESCs, potentially suggesting that these nanoparticles do not hamper cell membrane integrity. Cell apoptosis was analyzed by fluorescence‐activated cell sorting (FACS) (Figure S2C, Supporting Information); no obvious change in the apoptosis rate was identified in mESCs incubated with LDH at all doses when compared with those incubated in LIF+. The proliferation of nanoparticle‐treated mESCs was then evaluated by an EdU proliferation assay (Figure S2D, Supporting Information). The fluorescence intensities of EdU demonstrated no significant difference among both groups. Moreover, the percentage of proliferating cells under different conditions was quantified using the EdU Cell Proliferation Kit with Alexa Fluor 594 followed by FACS. As shown (Figure S2E, Supporting Information), no significant differences were observed under the different conditions for both groups. The above data confirmed the biocompatibility of the MgFe‐LDH and MgAl‐LDH nanoparticles and their suitability for mESC culture.

### 2.3 MgFe‐LDH Is Superior to MgAl‐LDH in Supporting mESC Self‐Renewal

**Figure 1:**
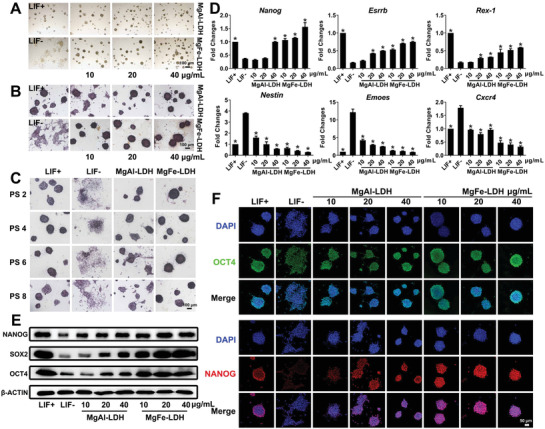
MgFe‐LDH is superior to MgAl‐LDH in supporting mESC self‐renewal. A) Morphology of clones observed via bright‐field microscopy. B) Representative images of mESCs cultured with nanoparticles and stained with ALP after 3 d of culture. C) ALP staining of mESCs treated with 20 µg mL^−1^ MgAl‐LDH or MgFe‐LDH at the indicated passage. D) qPCR analysis of key pluripotency genes (*Nanog*, *Esrrb*, and *Rex‐1*) and key differentiation genes (*Nestin*, *Eomes*, and *Cxcr4*). * represents *p* < 0.05, when compared to the LIF‐ treatment. E) Protein levels of NANOG, SOX2, and OCT4 in mESCs quantified by western blot. * represents *p* < 0.05, when compared to the LIF‐group. F) Representative confocal microscopy images of mESCs stained with OCT4 (green), NANOG (red), and DAPI (blue) accompanied by various treatments.

First, the biofunction of MgFe‐LDH or MgAl‐LDH in mESC culture was investigated by observing the clone morphology in each group. As depicted in **Figure** **1**A, the mESCs in the LIF+ group had a round shape, whereas most clones in the LIF‐ group were fragmented, presenting an irregular shape and undefined borders, considered typical characteristics of pluripotency loss. Interestingly, both MgFe‐LDH and MgAl‐LDH were able to accelerate the self‐renewal capabilities of the mESCs, in the absence of LIF. This was especially the case with MgFe‐LDH that effectively replaced the role of LIF in retaining clone morphology in a concentration‐dependent manner. It is worth mentioning that the clones in the MgFe‐LDH group were rounder than those in the MgAl‐LDH group at the same concentration as well as those in the LIF+ group. Colony circularity was quantified under different conditions using the ImageJ software along with the following formula: circularity = 4 × pi × area÷(perimeter squared). The circularity values equal to 1 indicate a perfect circle, whereas smaller values suggest an elongated shape in the mESCs. As shown (Figure S3A, Supporting Information), the LDH‐treated mESCs exhibited a significantly higher circularity index than the LIF‐ group, equaling or surpassing the LIF+ group. Furthermore, the circularity of the MgFe‐LDH group was greater than that of the MgAl‐LDH group at the same concentration, which supports the results of Figure 1A. To better characterize the clones in each treatment, alkaline phosphatase (ALP) staining was used to determine the pluripotency state of the mESCs. As depicted (Figure 1B), high ALP activity was detected in the LIF+ group (free of MEF only), whereas the LIF‐ group (free of MEF and LIF) demonstrated spontaneous differentiation and reduced ALP activity. Compared with the MgAl‐LDH group, the clones in the MgFe‐LDH group possessed higher ALP activity at the same concentration, indicating that MgFe‐LDH is superior to MgAl‐LDH in maintaining mESC self‐renewal. According to the quantification data (Figure S3B, Supporting Information), the MgFe‐LDH group showed a relatively darker color post ALP staining at all the tested concentrations, indicating enhanced ALP activity. However, the same level was only attained at 40 µg mL^−1^ for MgAl‐LDH. In order to determine the possible application of nanoparticles in long‐term cell culture, mESCs were cultured to passages 8 (PS8) in culture media containing MgFe‐LDH or MgAl‐LDH. Bright‐filed images (Figure S3C, Supporting Information) revealed that 10 µg mL^−1^ MgFe‐LDH was not able to support the long‐term cell culture during passaging and cells in this group showed obvious differentiation tendencies at PS6 and PS8. Thus, it was deduced that 10 µg mL^−1^ MgFe‐LDH is not suitable for mESCs culture and 20 µg mL^−1^ MgFe‐LDH was applied for further experiments. mESCs at passages 2, 4, 6, and 8 were stained with ALP (Figure 1C), and mESCs in the LIF‐ group were observed to be in a highly spontaneous differentiation stage in each passage. For every passage, mESCs in the MgFe‐LDH group always possessed higher ALP activity than the MgAl‐LDH group, which was particularly obvious at passages 8, when clones in the MgAl‐LDH group showed a clear differentiation trend. For long‐term culture (Figure S3D, Supporting Information), quantitative analysis results suggesting that the MgFe‐LDH group also exhibited significantly higher ALP activity in comparison with the MgAl‐LDH at all passages. The results of the clone morphology and ALP staining assessment were consistent and indicated that MgFe‐LDH is superior to MgAl‐LDH in maintaining mESC self‐renewal, and can be considered roughly the same as LIF treatment. Furthermore, total RNA was isolated and the messenger Ribonucleic Acid (mRNA) expression levels were analyzed via Real‐time Quantitative polymerase chain reaction (qPCR) (Figure 1D), as were pluripotency markers (*Nanog*, *Esrrb*, and *Rex‐1*) and differentiation markers (*Nestin*, *Eomes*, and *Cxcr4*). Remarkably, the expression of *Nanog*, *Esrrb*, and *Rex‐1* in the LIF‐ group was significantly lower than that in the LIF+ group. The nanoparticle‐treated group showed gene expression in a concentration‐dependent manner. The MgFe‐LDH group displayed higher *Nanog*, *Esrrb*, and *Rex‐1* expression levels than the MgAl‐LDH group at the same dose, indicating that MgFe‐LDH could better promote the self‐renewal of mESCs. On the other hand, the expression of differentiation‐related markers (*Nestin*, *Eomes*, and *Cxcr4*) in the MgFe‐LDH group was similar to that of the LIF+ group, and significantly lower than that in the MgAl‐LDH group at the same concentration, confirming that MgFe‐LDH was more effective in sustaining the self‐renewal of mESCs than MgAl‐LDH. Protein expression was also measured simultaneously (Figure 1E), and the western blot (WB) analysis clearly indicated that NANOG, organic cation/carnitine transporter4 (OCT4), and SRY‐box transcription factor 2 (SOX2) expression was significantly attenuated in the LIF‐ group when compared with that in the LIF+ group. Meanwhile, MgAl‐LDH and MgFe‐LDH treatments both led to a dose‐dependent activation of NANOG, OCT4, and SOX2 as compared with that in the LIF‐ group, in which the MgFe‐LDH group exhibited higher protein expression than the MgAl‐LDH group at all doses. Interestingly, the protein expression in the MgFe‐LDH group at 20 as well as 40 µg mL^−1^ were equal to, and even slightly higher than that in the LIF+ group. Furthermore, the qPCR and WB results were verified by the immunofluorescence analysis for the OCT4 and NANOG proteins (Figure 1F). It was observed that the expression of OCT4 and NANOG was greatly downregulated upon LIF withdrawal, whereas MgFe‐LDH and MgAl‐LDH treatment enhanced the fluorescence intensity of OCT4 and NANOG. Notably, the fluorescence intensity in the MgFe‐LDH group was stronger than that in the MgAl‐LDH group, at the same dose. To determine what happens if MgFe‐LDH or MgAl‐LDH treatment is ceased, mESCs were initially cultured in MgFe‐LDH and MgAl‐LDH culture systems for two passages. When the cells were passaged to passages 3 (PS3), the nanoparticles were withdrawn from the culture system for 3 d. Consequently, it could be clearly observed that the mESCs indeed lost their stem cell characteristics (Figure S3E, Supporting Information). Collectively, these results (Figure 1 and Figure S3, Supporting Information) indicate that MgFe‐LDH nanoparticles are superior to MgAl‐LDH nanoparticles in maintaining mESC self‐renewal and can be a satisfactory alternative for LIF in mESC culture.

### 2.4 MgFe‐LDH Surpasses MgAl‐LDH in Supporting the Pluripotency of mESCs

**Figure 2:**
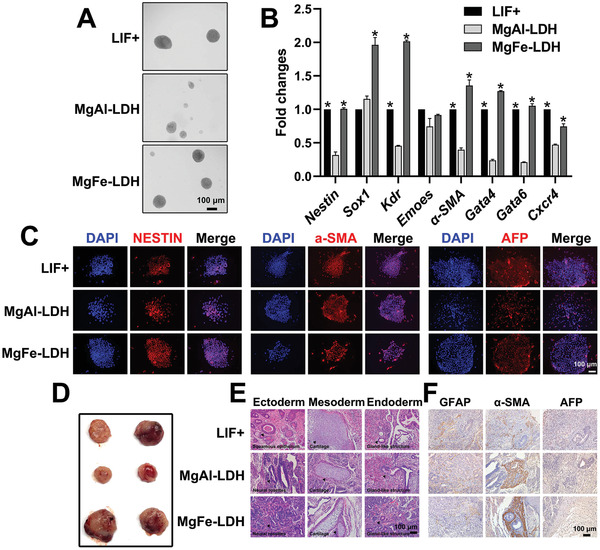
MgFe‐LDH is superior to MgAl‐LDH in supporting the pluripotency of mESCs. A) The embryoid bodies differentiated from P1 mESCs treated with nanoparticles at day eight of differentiation. B) The relative mRNA expression of three germ layer‐related genes during embryoid bodies differentiation at d8 was measured by qPCR: *Nestin* and *Sox1* in the ectoderm; *Kdr*, *Emoes*, and *α‐SMA* in the mesoderm; as well as *Gata4*, *Gata6*, and *Cxcr4* in the endoderm. * represents *p* < 0.05, when compared to the MgAl‐LDH treatment. C) The embryoid bodies were transferred to a gelatin precoated confocal dish for differentiation for another 4 d following which the presence and localization of NESTIN (ectoderm), *α*‐SMA (mesoderm), and AFP (endoderm) were determined by immunofluorescence. D) Teratoma formation assay for assessing pluripotency of P3 mESCs treated with nanoparticles. E) Histological analysis by HE staining of teratomas tissues derived from P3 mESCs treated with nanoparticles, to analyze the morphological features of the three germ layers. Arrowheads indicate these morphological features. F) Immunohistochemistry analysis of teratomas sections.

Another important feature of mESCs is their potential for differentiating into three germ layers capable of assembling various tissues or organs. Embryoid bodies (EBs) in vitro and teratomas in vivo are commonly used to investigate the differentiation potential of mESCs. EBs formation was more regular in the MgFe‐LDH group than in the MgAl‐LDH group, similar to the behavior in the LIF+ group (**Figure** **2**A). Subsequently, qPCR was conducted to detect gene expression in the different germ layers formed in the EBs (Figure 2B). Although the marker genes of the three germ layers (*Nestin*, *Sox1*, *Kdr*, *Emoes*, alpha smooth muscle actin (α‐SMA), *Gata4*, *Gata6*, and *Cxcr4*) were present in all the groups, the expression levels in the MgFe‐LDH group were significantly higher than those in the MgAl‐LDH group. In addition, we found that some were even higher than those in the LIF+ group, indicating that MgFe‐LDH nanoparticles were able to give rise to the three germ layers more efficiently than MgAl‐LDH and LIF. The EBs were then allowed to remain adherent for differentiation for a period of four more days, and the expression of the three germ layer markers containing NESTIN (ectoderm), *α*‐SMA (mesoderm), and alpha fetoprotein (AFP) (endoderm) was tested by immunostaining. The results indicated that EBs in all the groups could differentiate into different types of cells in all three germ layers (Figure 2C). Furthermore, mESCs treated with nanoparticles were subcutaneously injected into nude mice to yield in vivo differentiated teratomas (Figure 2D). It was clearly observed that the teratoma's size in the MgFe‐LDH group was significantly larger than that in the MgAl‐LDH group, indicating that the MgFe‐LDH‐treated mESCs possessed stronger differentiation ability. Hematoxylin‐eosin (HE) staining of teratomas was performed in order to visualize the structural features of the three germ layers (Figure 2E). Similar to the LIF+ group, the structural features of the three germ layers can also be clearly observed in the MgFe‐LDH and MgAl‐LDH groups. Further immunohistochemical analysis of tissue sections revealed that teratomas from all treatments were capable of expressing glial fibrillary acidic protein (GFAP) (ectoderm), AFP (endoderm), as well as *α*‐SMA (mesoderm) (Figure 2F).

### 2.5 Transcriptomic and Proteomic Analyses

To further compare the role of MgAl‐LDH and MgFe‐LDH nanoparticles in regulating mESC gene expression patterns, we examined the transcriptomes of mESCs treated with MgFe‐LDH and MgAl‐LDH nanoparticles via RNA sequencing (RNA‐seq). Total RNA from 12 samples (three biological replicates for each treatment) were quantified to obtain an average of 152.7, 118.9, 88.0, and 123.0 µg RNA for the LIF+, LIF‐, MgAl‐LDH, and MgFe‐LDH groups, respectively (Figure S4A, Supporting Information). Subsequently, the transcriptomic analysis was performed, and principal component analysis (PCA) results suggested that the three replicates in each group were distributed in a concentrated manner (Figure S4B, Supporting Information). Kyoto encyclopedia of genes and genomes (KEGG) pathway analysis further confirmed that differentially expressed genes (DEGs) were enriched in many biological processes, including focal adhesion and signaling pathways regulating pluripotency (Figure S4C, Supporting Information). A heatmap of clustering analysis found that compared to the LIF‐ and MgAl‐LDH groups, the gene expression profile of the LIF+ group was more similar to that of the MgFe‐LDH group (Figure S4D, Supporting Information). In addition, the pathways related to signal transduction or transport and catabolism were observed to be significantly different when analyzing DEGs between MgAl‐LDH and MgFe‐LDH.

Protein was extracted and quantified as 222.5, 231.9, 182.5, and 238.3 µg on average for the LIF+, LIF‐, MgAl‐LDH, and MgFe‐LDH groups, respectively (Figure S5A, Supporting Information). The proteomic results of PCA analysis confirmed that the LIF‐ group was most similar to the MgAl‐LDH group, followed by the MgFe‐LDH and LIF+ groups (Figure S5B, Supporting Information). Furthermore, functional classification provided by Eukaryotic orthologous groups analysis revealed that differentially expressed proteins (DEPs) between the MgAl‐LDH and MgFe‐LDH groups were mostly enriched in signal transduction mechanisms as well as inorganic ion transport and metabolism (Figure S5C, Supporting Information). The top enriched gene ontology (GO) terms were binding, cell, and cellular processes for molecular function, cellular component, and biological process, respectively (Figure S6, Supporting Information).

### 2.6 Combined Transcriptomic and Proteomic Analysis

**Figure 3:**
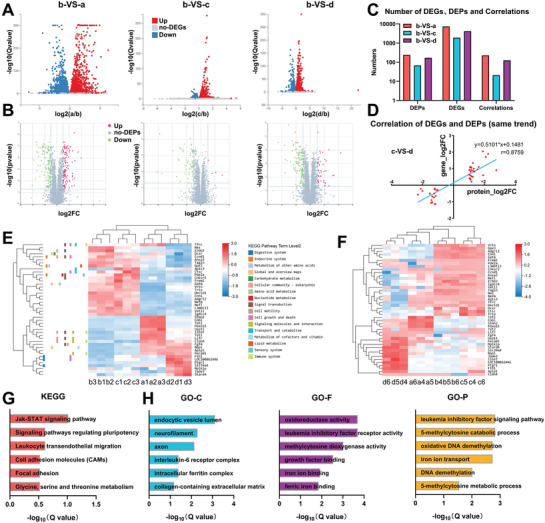
LIF signaling pathway, DNA demethylation, and iron ion transport differs significantly between the MgFe‐LDH and MgAl‐LDH groups, as identified via combined transcriptomic and proteomic analysis. A) Volcano plot of DEGs. B) Volcano plot of DEPs. C) The number of DEGs, DEPs, and correlations were quantified. a, b, c, and d represent the LIF+, LIF‐, MgAl‐LDH, and MgFe‐LDH groups, respectively. D) Correlation analysis of 44 DEGs and DEPs that had similar trends between c) the MgAl‐LDH and d) MgFe‐LDH groups. E) Heatmap of these 44 DEGs from correlation analysis, colors indicating relative expression. F) Clustering analysis of these 44 DEPs from the correlation analysis. G) KEGG pathway analysis of these 44 DEGs. H) GO enrichment analysis of these 44 DEGs.

Additionally, combined transcriptomic and proteomic analysis was performed, since multiple omics analysis lowers the rate of false positives induced by single omics analysis. A volcano plot was used to make a clear comparison between the groups and further facilitate the screening of DEGs (**Figure** **3**A) as well as DEPs (Figure 3B). The number of DEGs, DEPs, and correlations were counted (Figure 3C). With regard to the expression pattern of genes and proteins, the LIF+ group shared the most similar expression profile with that of the MgFe‐LDH group, amongst all the other groups. The genes with the same expression trend at the transcriptomic and proteomic level in c‐VS‐d (MgAl‐LDH‐VS‐MgFe‐LDH) were listed. Of these, 44 genes and proteins shared the same change trend, wherein 26 were upregulated, 18 were downregulated in the MgFe‐LDH group, and the linear regression equation was *y* = 0.5101*x* + 0.1481, *r* = 0.8759 (Figure 3D). Moreover, the clustering analysis of these 44 genes was performed (Figure 3E) and it was clearly observed that the LIF‐ group was most similar to the MgAl‐LDH group, whereas the LIF+ group was similar to the MgFe‐LDH group. Concurrently, annotations revealed that these DEGs were primarily enriched in signal transduction pathways. In addition, the clustering analysis of the above 44 proteins was performed (Figure 3F). In consistent with the results in Figure 3E, the expression pattern of the MgFe‐LDH group differed significantly from that of the MgAl‐LDH and LIF‐ groups but was similar to that of the LIF+ group. KEGG Pathway and GO enrichment analysis was further performed for these 44 genes, wherein the Jak‐STAT signaling pathway and signaling pathways regulating pluripotency were regarded as two significantly enriched pathways (Figure 3G). GO annotation was classified into three categories (Figure 3H): GO‐C was related to the interleukin‐6 receptor complex and intracellular ferritin complex, GO‐F was associated with oxidoreductase activity, leukemia inhibitory factor receptor activity, methylcytosine dioxygenase activity, iron ion binding, and ferric iron binding, and GO‐P was related to the leukemia inhibitory factor signaling pathway, 5‐methylcytosine catabolic process, oxidative DNA demethylation, iron ion transport, DNA demethylation, and 5‐methylcytosine metabolic process. In summary, GO analysis identified leukemia inhibitory factor receptor activity, oxidative DNA demethylation, and iron ion transport to include highly enriched terms, which further facilitated our investigation.

### 2.7 MgFe‐LDH Nanoparticles Maintain the Pluripotency of mECSs via LIFR/PI3K/AKT, LIFR/JAK/STAT3, and phospho‐signal transducer and activator of transcription 3 (p‐STAT3)/TET Pathways

As per the results of the KEGG pathway enrichment analysis (Figure 3G), we identified LIFR and IL6ST (also known as GP130) in the signaling pathways regulating pluripotency. In order to understand the biological and molecular importance of MgFe‐LDH nanoparticles in mESC culture and to determine whether MgFe‐LDH nanoparticles could function via LIFR and GP130 as LIF does, LIFR inhibitor EC359 was administered to each group at a concentration of 150 × 10^−6^ m. EC359 is capable of directly interacting with LIFR such that the LIF/LIFR interaction is effectively blocked.^[^ ^31^, ^32^ ^]^

**Figure 4:**
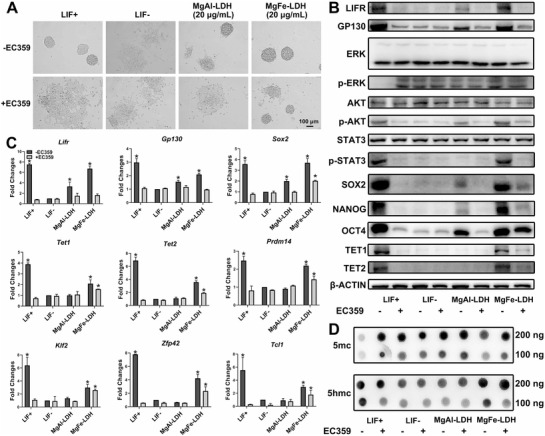
MgFe‐LDH nanoparticles maintain the pluripotency of mECSs via LIFR/PI3K/AKT, LIFR/JAK/STAT3, and p‐STAT3/TET pathways. A) Bright‐field images of mESCs cultured for 3 d in media containing LIF/LIFR inhibitor EC359. B) Western blot was applied to measure protein expression changes in mESCs cultured for 3 d on gelatin, with or without EC359, subjected to the requisite treatment. C) Analysis of changes in mRNA expression following EC359 treatment via qPCR. * represents *p* < 0.05, when compared to the LIF‐group. D) Global 5mc and 5hmc levels detected by dot blot.

Morphology observation indicated that MgFe‐LDH‐treated mESCs could remain in a partially undifferentiated state, even after EC359 treatment. However, the mESCs in the other groups, including LIF+ and MgAl‐LDH, could not maintain pluripotency (**Figure** **4**A). Moreover, addition of EC359 significantly decreased the LIFR‐mediated activation of p‐STAT3 and phospho‐protein kinase B(p‐AKT) whereas increased phospho‐extracellular signal‐regulated kinase (p‐ERK) expression in the LIF+ group (Figure 4B), suggesting that LIF/LIFR interacts with LIFR/JAK/STAT3, LIFR/PI3K/AKT, and LIFR/SHP2/MAPK signaling pathways to maintain pluripotency, as previously reported. Compared to the LIF‐ group, MgAl‐LDH treatment only increased the expression of p‐AKT,^[^ ^24^ ^]^ whereas MgFe‐LDH treatment activated both p‐AKT as well as p‐STAT3. In addition, EC359 administration downregulated p‐AKT expression in the MgAl‐LDH group, and p‐AKT as well as p‐STAT3 expression in the MgFe‐LDH group. These results indicate that LIFR/PI3K/AKT is a common pathway for MgAl‐LDH and MgFe‐LDH to maintain mECSs pluripotency; however, LIFR/JAK/STAT3 could only be activated by MgFe‐LDH. It was also noticed that SOX2, NANOG and OCT4 were expressed at a relatively high level in the LIF+ group but at a relatively low level in the LIF‐ group. Both MgFe‐LDH and MgAl‐LDH treatment increased increase SOX2, NANOG, and OCT4 expression compared with the LIF‐ group. Notably, MgFe‐LDH was more effective than MgAl‐LDH in promoting their expression. The addition of EC359 robustly decreased SOX2, NANOG, and OCT4 expression in the LIF+ and MgAl‐LDH groups to the levels observed in the LIF‐ group. EC359 reduced their expression in the MgFe‐LDH group as well; however, unlike the MgAl‐LDH and LIF+ groups, the expression levels in the MgFe‐LDH group was still significantly higher than in the LIF‐ group, following EC359 addition. Thus, alternative mechanisms might exist in the regulation of mESC pluripotency by MgFe‐LDH, which differs significantly from LIF or MgAl‐LDH.

### 2.8 DNA Demethylation and TET1/2 Regulation Are Involved in the MgFe‐LDH Group

GO analysis demonstrated the differences in iron ion binding or transport as well as DNA demethylation between the MgFe‐LDH and MgAl‐LDH groups. Epigenetic mechanisms involving DNA demethylation are also closely associated with ESC pluripotency state, while DNA demethylation is a TET‐mediated process. Thus, TET1/2 was selected from the GO analysis. TET1/2 expression was relatively high in the LIF+ group and relatively low in the LIF‐ group, while it was increased upon treatment with MgFe‐LDH but not with MgAl‐LDH. Coadministration of EC359 resulted in decreased TET1/2 levels in the LIF+ group, proving that TET1/2 was directly regulated by p‐STAT3, as reported in a previous study.^[^ ^33^ ^]^ With regard to the MgFe‐LDH + EC359 group, EC359 decreased TET1/2 levels, but they were still significantly higher than LIF‐ group. This was not in accordance with the expression pattern of p‐STAT3; thus, we propose that the TET or TET‐regulated process is not affected only by p‐STAT3 in the MgFe‐LDH group.

Furthermore, qPCR was used to measure *Lifr*, *Gp130*, *Sox2*, *Tet1*, and *Tet2* expression (Figure 4C), which was in consistent with the protein expression. TET1/2 regulated pluripotency‐related *Prdm14*, *Klf2*, *Dppa3*, *Zfp42*, as well as *Tcl1* expression. While also following the *Tet* expression pattern,^[^ ^34^ ^]^ EC359 treatment decreased the expression of these genes in the LIF+ and MgAl‐LDH groups to that of the levels found in the LIF‐ group. Remarkably, EC359 did not entirely decrease the expression of these genes in the MgFe‐LDH group, which was still significantly higher than that in the LIF‐ group.

It has been demonstrated that TET can convert 5mc to 5hmc, and further derivatives, in an Fe^2+^‐ and *α*‐ketoglutarate (*α*‐KG)‐dependent manner.^[^ ^34^, ^35^, ^36^, ^37^ ^]^ TET1 and TET2 proteins share a conserved cysteine‐rich region, and the dioxygenase motif is involved in *α*‐KG as well as Fe^2+^ binding.^[^ ^35^, ^38^ ^]^ The balance between the 5mc and 5hmc levels is correlated to the balance between pluripotency and differentiation. To this end, dot blot was applied to quantify global 5mc and 5hmc levels (Figure 4D). A striking global increase in 5hmc levels was observed in the LIF+ and MgFe‐LDH groups, but not in the MgAl‐LDH group. Global levels of 5hmc in the LIF+ group were decreased after EC359 treatment; the MgFe‐LDH group exhibited a decreasing trend but their levels was still significantly higher than that in the LIF‐ group. The trend for 5mc level was opposite to that of the 5hmc level.

The partially undifferentiated state of the MgFe‐LDH and EC359 groups could be due to the specificity and effectiveness of this inhibitor EC359. To better study the mechanism, we used si‐Lifr to knockdown LIFR (Figure S7, Supporting Information). All results in Figure S7 in the Supporting Information proved a similar conclusion to the one obtained from Figure 4. These results undisputedly suggest that MgFe‐LDH nanoparticles can affect the maintenance of DNA demethylation landscapes in mESCs, as indicated by combined transcriptomic and proteomic analysis. Further, TET abundance and TET‐mediated 5hmc conversion was not only regulated solely by p‐STAT3 in the MgFe‐LDH group; thus, there must exist other mechanisms affecting the self‐renewal process.

### 2.9 Fe^2+^ Supplied by MgFe‐LDH Enhances TET Expression

**Figure 5:**
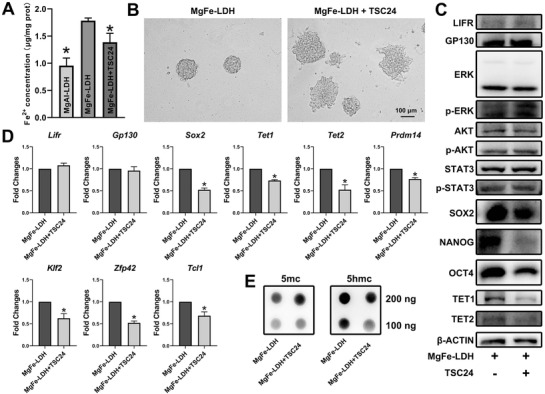
Fe^2+^ supplied by MgFe‐LDH enhances TET expression. A) Fe^2+^ concentration in the MgAl‐LDH, MgFe‐LDH, and MgFe‐LDH+TSC24 groups. B) Morphological changes in mESCs after Fe^2+^ chelator TSC24 was added to the MgFe‐LDH group. C) Changes in protein expression following TSC24 treatment, detected by western blot. D) *Tet1/2* and *Tet1/2* regulated pluripotency‐related gene expression following TSC24 administration. * represents *p* < 0.05, when compared to the MgFe‐LDH group. E) Detection of global 5mc and 5hmc levels following TSC24 administration.

GO analysis reinforced that mechanisms of iron ion transport and binding differ between MgFe‐LDH and MgAl‐LDH. It is well known that TET activities are Fe^2+^ dependent; also, it has been reported that Fe^2+^ could enhance TET1/2 expression.^[^ ^38^, ^39^ ^]^ We speculated that MgFe‐LDH nanoparticles could provide additional Fe^2+^, contributing to the functioning of TET and pluripotency maintenance in mESCs.^[^ ^40^ ^]^ In order to determine the role of Fe^2+^ in this entire process, a set of experiments were designed to assess the MgAl‐LDH, MgFe‐LDH, and MgFe‐LDH+TSC24 (a specific Fe^2+^ chelator) groups. To this end, Fe^2+^ concentration was detected in each group in the order of 0.95, 1.78, and 1.38 µg mg^−1^ protein for the MgAl‐LDH, MgFe‐LDH, and MgFe‐LDH+TSC24 groups, respectively (**Figure** **5**A). MgFe‐LDH provided supplemental Fe^2+^, while TSC24 decreased the Fe^2+^ concentration in the MgFe‐LDH group. Images of morphology change confirmed that TSC24 treatment could affect undifferentiated mESCs maintained in MgFe‐LDH (Figure 5B), implying that Fe^2+^ plays a vital role in sustaining mESC pluripotency. Additionally, western blot, qPCR as well as dot blot analyses were performed in order to further investigate the role of Fe^2+^ in the MgFe‐LDH group. TSC24 decreased TET1/2, SOX2, NANOG, and OCT4 expression in the MgFe‐LDH group (Figure 5C) but exhibited no such effect on LIFR, GP130, p‐ERK, p‐AKT, and p‐STAT3 expression. This result was further confirmed by the qPCR data (Figure 5D), in which *Prdm14*, *Klf2*, *Dppa3*, *Zfp42*, and *Tcl1* gene expression demonstrated the same trend as *Tet1/2* after TSC24 treatment. Subsequently, the 5mc and 5hmc levels in each group were measured (Figure 5E), wherein TSC24 was observed to downregulated 5hmc levels in the MgFe‐LDH group, as expected. Our data show that Fe^2+^ supplied by MgFe‐LDH could enhance TET1/2 expression, along with 5hmc levels and pluripotency‐related genes regulated by TET1/2, which ultimately benefit for the maintenance of mESC pluripotency.

It has been reported that nanomaterials can affect cell fate by interacting with cell membrane receptors.^[^ ^41^, ^42^ ^]^ Positively charged MgFe‐LDH was observed to interact with the negatively charged cell membrane easily. In addition, to determine if there was any interaction between the nanoparticles and LIFR, we labeled LDH with fluorescein isothiocyanate (FITC) and LIFR with LIFR antibody. As shown (Figure S8A, Supporting Information), LIFR was expressed and located in the cell membrane in accordance with previous findings, whereas both MgFe‐LDH and MgAl‐LDH, with a green colored signal, showed obvious colocalization with LIFR in the cell membrane, indicating that LDH could interact with LIFR. Furthermore, the fluorescence intensity of LIFR in the MgAl‐LDH group was weaker than that in the MgFe‐LDH and LIF+ groups, consistent with the results in Figure 4B,C, which depict the protein and mRNA expression of LIFR. Moreover, the single function of Fe^2+^ was investigated in order to study the effect of a single component (nanoparticles or ions) on sustaining self‐renewal. The function of nanoparticles has already been examined (Figure 5), wherein they were found to partially sustain cell self‐renewal. Hence, for ions, we investigated the function of ferric nitrate (used in MgFe‐LDH fabrication) in supporting mESC self‐renewal. As demonstrated (Figure S8B,C, Supporting Information), only a high level of ferric nitrate treatment (120 × 10^−6^ m, approximately corresponding to 40 µg mL^−1^ MgFe‐LDH) could slightly mimic the function of MgFe‐LDH. Thus, it can be concluded that ions are also important, but only have a limited effect in supporting mESC self‐renewal. In summary, nanoparticles were observed to be more efficient than ions in our study. Furthermore, Fe^2+^ release from MgFe‐LDH was detected, and it was found that after the administration of LDH, the intracellular Fe^2+^ concentration in the MgFe‐LDH group was always higher than that in the MgAl‐LDH group (Figure S8D, Supporting Information), suggesting that the MgFe‐LDH group could provide additional Fe^2+^ for mESC culture as compared to the MgAl‐LDH group. Contrastingly, EC359 or TSC24 weakened the functioning of MgFe‐LDH to some extent (Figures 4 and 5). Moreover, when MgFe‐LDH was co‐administered with EC359 and TSC24, all the cells underwent a differentiation state similar to that of the LIF‐ groups (Figure S8E, Supporting Information), proving that MgFe‐LDH exerts its function only through these two mechanisms.

## 3 Discussion

In this study, we reported the use of MgFe‐LDH nanoparticles in mESC culture for the first time. We conclusively proved the capacity of MgFe‐LDH to maintain the self‐renewal and pluripotency of mESCs in the absence of MEF and LIF. Compared to other inorganic nanoparticles, LDH has many advantages: 1) It can be easily synthesized, 2) chemically well‐defined, 3) extremely low toxicity, and 4) controllable chemical and physical properties. M^2+^ (divalent metal ions) in LDH can be diverse from Mg^2+^, Ni^2+^, Zn^2+^, Cu^2+^, Co^2+^, Mn^2+^, and Ca^2+^; M^3+^ (trivalent metal ions) can be chosen from Al^3+^, Cr^3+^, Fe^3+^, Ca^3+^, Co^3+^, Sc^3+^, V^3+^, and Mn^3+^. This allows researchers to optimize LDH according to the requirements specific to their studies. Various permutations and combinations of M^2+^ and M^3+^ in LDH endow limitless potential for its applications. In our study, we selected Al^3+^ and Fe^3+^ to make a comparison, for which we synthesized MgFe‐LDH and MgAl‐LDH nanoparticles. As the second highest trace element in the human body, iron is an essential molecule for the human body. Iron deficiency is one of the most important causes of anemia, immune dysfunction, and axon maturation.^[^ ^43^ ^]^ However, aluminum and its compounds are not as harmful to humans as compared to their significant beneficial contributions, and it is well known that requirements and tolerance of aluminum in mice are far below that of iron.

According our study, MgFe‐LDH was observed to be more advantageous than MgAl‐LDH in maintaining mESC pluripotency, indicated by rounder clone morphology, higher activity of ALP as well as increased expression levels of self‐renewal related genes and proteins. In addition, EBs in the MgFe‐LDH group possess better spontaneous differentiation capabilities than those in the MgAl‐LDH group. Further teratoma formation experiments confirmed that the volume of teratomas in the MgFe‐LDH group was larger than that in the MgAl‐LDH group. However, it should be noted that biocompatible MgFe‐LDH and MgAl‐LDH nanoparticles did not affect mESC proliferation, and they had limited differences in their physical properties. Hence, the underlying molecular mechanisms should be investigated in detail.

**Figure 6:**
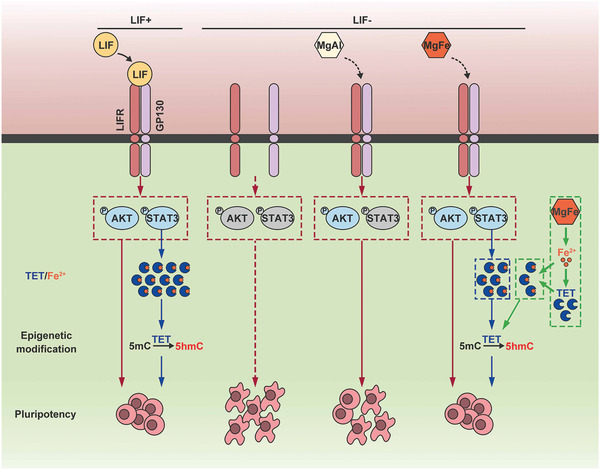
Schematic representation of the functional mechanisms of MgFe‐LDH nanoparticles in regulating mESC pluripotency. MgFe‐LDH nanoparticles can activate the LIFR/PI3K/AKT and LIFR/JAK/STAT3 signaling pathways, resulting in downstream TET1/2 expression promoted by the activated p‐STAT3 thus controlling the expression of pluripotency‐related genes and DNA demethylation. In addition, Fe^2+^ released from MgFe‐LDH nanoparticles also proves beneficial in enhancing TET expression. All the above mechanisms ultimately make MgFe‐LDH an excellent substitute for LIF in maintaining mESC pluripotency.

Previous studies have confirmed that the expression levels of mRNA and their corresponding proteins may be inconsistent. Thus, the integration and analysis of the data from the two omics will be more conducive to the study of gene expression patterns. Combined transcriptomic and proteomic analysis can not only reveal the essence of life activities at protein and transcription levels but also explain the interaction between them.^[^ ^44^ ^]^ The transcriptomic and proteomic data were initially analyzed separately, but uncertainty remained as to whether the mRNA and protein expression data corroborated. Therefore, combined transcriptomic and proteomic analysis was applied in our study, in order to specifically identify key pathways or mRNAs. The underlying mechanisms due to which MgFe‐LDH is superior to MgAl‐LDH as well as LIF in maintaining mESC pluripotency can be summarized as follows (**Figure** **6**): 1) MgFe‐LDH could maintain mESC pluripotency via LIFR/PI3K/AKT, similar to both MgAl‐LDH and LIF. 2) MgFe‐LDH also functions via LIFR/JAK/STAT3, which is common to LIF as well. 3) Importantly, TET1/2 abundance can be affected by the Fe^2+^ provided by MgFe‐LDH, except for the regulation of p‐STAT3.

The mechanisms under study further indicated that iron ions released from MgFe‐LDH also affected the mESC state. It has been previously demonstrated that iron homeostasis is crucial in several cellular activities, including proliferation, cell death, and differentiation.^[^ ^45^ ^]^ Moreover, growing evidence has revealed that iron homeostasis is of great importance in the regulation of stem cell pluripotency.^[^ ^27^ ^]^ Notably, iron can affect the self‐renewal and functioning of hematopoietic stem and progenitor cells.^[^ ^28^, ^46^ ^]^ Magnetic nanoparticles (MNPs) can be used to maintain mESCs in the absence of MEF.^[^ ^29^ ^]^ The intracellular depletion of iron leads to a rapid downregulation of NANOG and a dramatic decrease in the self‐renewal of human pluripotent stem cells (hPSCs), as well as spontaneous and nonspecific differentiation.^[^ ^26^ ^]^ In this study, we proposed that the iron ion provided by MgFe‐LDH proves beneficial to the abundance of TET1/2, thus aiding the maintenance of mESC pluripotency.

Altogether, the MgFe‐LDH nanoparticles in this study provided excellent support for the maintenance of mESC pluripotency under LIF‐free conditions. Moreover, our study provides remarkable insights into the significance of the effect of MgFe‐LDH nanoparticles on mESC cultures, along with the benefits of their consequent application in regenerative medicine and functional tissue engineering.

## 4 Experimental Section

##### Chemicals

Mg(NO_3_)_2_·6H_2_O, Al(NO_3_)_3_·9H_2_O, Fe(NO_3_)_3_·9H_2_O, NaOH, and KBr were purchased from Sinopharm Group Co. Ltd. (Shanghai, China). Dulbecco's modified eagle medium (DMEM), fetal bovine serum (FBS), Glutamax, nonessential amino acids (NEAA), sodium pyruvate (SP), penicillin and streptomycin, and trypsin were obtained from Thermo Fisher Scientific (MA, USA). LIF and Gelatin were purchased from Millipore (MA, USA). *β*‐mercaptoethanol was supplied by Sigma‐Aldrich (MO, USA). Antibodies against NESTIN, *α*‐SMA, AFP, GFAP, STAT3, and p‐STAT3 were bought from Abcam (Cambridge, England). LIFR, GP130, and TET2 antibodies were provided by Proteintech (Wuhan, China). TET1 antibody was bought from ABclonal (Wuhan, China). *β*‐ACTIN antibody was obtained from Bioss (Beijing, China). NANOG, SOX2, OCT4, ERK, p‐ERK, AKT, and p‐AKT antibodies were purchased from Cell Signaling Technology (CST) (MA, USA). 5mc and 5hmc antibodies were obtained from Active Motif (CA, USA). EC359 was obtained from MedChemExpress(NJ, USA). TSC24 was purchased from Sigma‐Aldrich. All small interfering Ribonucleic Acid (siRNAs) used in the study were synthesized by RiboBio (Guangzhou, China).

##### Synthetization and Characterization of MgAl‐LDH and MgFe‐LDH Nanoparticles

MgFe‐LDH and MgAl‐LDH nanoparticles were synthesized via a coprecipitation and subsequent hydrothermal protocol.^[^ ^24^ ^]^ Briefly, 1.538 g Mg(NO_3_)_2_·6H_2_O and 0.606 g Fe(NO_3_)_3_·9H_2_O dissolved in 20 mL ddH_2_O were added into stirring NaOH solution at 60 °C for 30 min. The obtained sediment was then undergoing a hydrothermal procedure at 100 °C for 16 h. After centrifugation and ddH_2_O washing, MgFe‐LDH nanoparticles were obtained. MgAl‐LDH nanoparticles were obtained by replacing 0.606 g Fe(NO_3_)_3_·9H_2_O with 0.75 g Al(NO_3_)_3_·9H_2_O.

TEM and SEM were performed to observe the morphology of nanoparticles. Copper net used in TEM was bought from Zhongjingkeyi (Beijing,China). The phase of MgFe‐LDH and MgAl‐LDH nanoparticles was detected using X‐ray diffraction (2*θ* ranging from 10° to 80 °Cu K*α*1), and the results were analyzed with Jade. FTIR spectrometer was applied to characterize the nanoparticles in the range of 500–4000 cm^−1^, using a standard KBr disk method (Nanoparticles/KBr = 1/200). Hydrodynamic diameters and Zeta potential were determined by the Malvern Nano Zetasizer series.

##### Cell Culture

Mouse embryonic stem cells (mESCs) were cultured on MEF in LIF‐containing medium under suitable conditions (5% CO_2_, 37 °C). mESCs maintenance medium consisted of 66 mL DMEM, 30 mL FBS, 1 mL Glutamax, 1 mL nonessential amino acids (NEAA), 1 mL SP, 1 mL penicillin and streptomycin, 0.1 × 10^−3^ m *β*‐mercaptoethanol, and 1000 units mL^−1^ LIF. mESCs were passaged every 2–3 d by incubation with 0.05% trypsin–ethylene diamine tetraacetic acid (EDTA) solution for 2 min, and the culture medium was refreshed daily.

##### CCK‐8 Test

CCK‐8 (APExBIO Technology, Houston) was used to characterize the cell growth of mESCs treated with nanoparticles according to the manufacturer's instructions. First, MEF were excluded from mESCs by a differential adherence method. Briefly, after trypsinization cells were collected and transferred to the original well for 20 min to allow MEF to attach to the plate; the supernatant was obtained, and the mESCs were seeded into gelatin pretreated 96‐well plates at a density of 1 × 10^3^ cells well^−1^ overnight in LIF‐containing medium. Wells with no cells but medium were served as the blank control group. Subsequently, the LIF‐contained culture medium was changed with 5, 10, 20, and 40 µg mL^−1^ MgFe‐LDH and MgAl‐LDH nanoparticles contained medium free of LIF every day. 24 or 48 h later, mESCs were incubated with 10 µL CCK‐8 solution for additional 3 h at 37 °C. The absorbance was recorded at 450 nm by a microplate reader (Thermo Fisher Scientific Inc.), and the cell viability in each group was calculated accordingly, as compared with the LIF+ group (100%).

##### Lactate Dehydrogenase Release Assay

Lactate dehydrogenase assay kit (Beyotime Biotechnology, Shanghai) was used to investigate the integrity of cell membrane. The cells were seeded overnight followed by nanoparticle administration for 24 or 48 h. Subsequently, 100 µL supernatant was transferred to a fresh 96‐well plate containing 100 µL working solution at room temperature for 30 min. The absorbance was recorded at 490 nm with a microplate reader.

##### Cell Apoptosis Detection

Cell apoptosis was investigated with an Annexin V‐FITC/propidium iodide (PI) kit (KeyGen, Nanjing). mESCs were treated with 5, 10, 20, and 40 µg mL^−1^ MgFe‐LDH or MgAl‐LDH nanoparticles for three continuous days, and the nanoparticle‐containing medium was refreshed every day. Cells cultured with LIF were served as the control group. After trypsinization, cells were incubated with 500 µL binding buffer (including 5 µL Annexin V‐FITC and 5 µL PI) in dark for 10 min. After washing with phosphate buffer saline (PBS), cell apoptosis was detected by flow cytometry.

##### EdU Detection

EdU detection kit (Yeasen Biotech, Shanghai) was applied to validate cell proliferation. Cells were seeded without MEF at a density of 2 × 10^4^ mL^−1^ on gelatin‐coated confocal dishes overnight. The medium was refreshed with nanoparticle‐containing medium every day for 3 d. mESCs were mixed with 10 × 10^−6^ m EdU solution for 3 h prior to 4% paraformaldehyde (PFA) fixation. 1 mL Click‐iT working buffer was used to react with EdU for 30 min. Phalloidin‐FITC and 4',6‐diamidino‐2‐phenylindole (DAPI) were applied to label cytoskeleton and cell nucleus, respectively. EdU Cell Proliferation Kit with Alexa Fluor 594 (LMAIBio, Shanghai) was used to quantify the percentage of proliferating cells in different conditions. mESCs were mixed with Click‐iT working buffer for 30 min, and Hoechst was then used to label cell nucleus. FACS was applied then.

##### Alkaline Phosphatase (ALP) Staining

mESCs seeded on 6‐well plates overnight were divided into several groups, including LIF+, LIF‐, MgAl‐LDH, and MgFe‐LDH groups. 3 d later, cells were observed directly or after ALP staining (Alkaline Phosphatase, Sidansai Biotechnology, Shanghai) via bright‐filed microscopy. ALP staining was conducted as follows: after 4% PFA fixation, cells were incubated with ALP working buffer for 15–30 min. To figure out the difference of long‐term culture of mESCs in MgAl‐LDH or MgFe‐LDH system as compared to LIF system, mESCs were passaged to passages 8. 20 µg mL^−1^ MgAl‐LDH and MgFe‐LDH nanoparticles were used during the whole process, and ALP staining was performed every two passage.

##### Western Blot

Protein for western blot was isolated using a protein extraction kit (Keygen, Nanjing). Briefly, cells with different treatments were collected, and 200 µL lysis buffer (including 2 µL phenylmethylsulfonyl fluoride (PMSF), 0.2 µL protease, and 2 µL phosphatase inhibitors) was used to lyse cells. Centrifuge at a speed of 12 000 rpm for 15 min to obtain the supernatant. Samples were quantified by butyleyanoacrylate (BCA) kit (Keygen, Nanjing), prior to boiled with loading buffer at 95 °C for 5 min. 20 µg protein were loaded for each sample, and separated by sodium dodecyl sulfate polyacrylamide gel electrophoresis (SDS‐PAGE) gels. Subsequently, protein was transferred to a polyvinylidene fluoride (PVDF) membrane, blocked in 5% w/v bovine serum albumin (BSA), and incubated with indicated primary antibodies overnight at 4 °C. The membranes were washed three times with tris buffered saline tween (TBST), incubated with secondary antibody at room temperature for 1 h, and washed three times by TBST. Electrochemiluminescence (ECL) kit (Millipore) was applied for chemiluminescence and the bands were observed with a Tanon chemiluminescence detection system.

##### qPCR

Total RNA was extracted from mESCs with TRIzol (Takara, Japan) reagent. 500 ng of RNA was used for cDNA conversion via Primer Script reverse transcriptase kit (Takara). Quantitative real‐time PCR was carried out using SYBR Premix (Takara) on the Q7 Flex Real‐Time PCR instrument. The list of primers (Sangon Biotech, China) is presented (Table S1, Supporting Information). A relative quantification (∆∆Ct) method was applied to calculate relative amounts of mRNA. *Gapdh* was served as the internal reference.

##### Immunofluorescence Staining

Cells treated with or without nanoparticles were fixed in 4% PFA for 10–20 min followed by permeabilizing in 0.25% Triton‐X100 for 10–20 min at the indicated times. After incubation with blocking buffer (normal goat serum and 0.3% Triton X‐100 in PBS) for 2 h, cells were then incubated with primary antibodies diluted in blocking buffer overnight at 4 °C. Subsequently, cells were washed and incubated with secondary antibodies for 2 h. DAPI (Sigma) was applied for nucleus staining.

##### Embryoid Bodies (EBs) Formation

mESCs cultured with 20 µg mL^−1^ MgAl‐LDH or MgFe‐LDH nanoparticles for 3 d were harvested and a total of 2 × 10^5^ cells were cultured in suspension without LIF in an ultra‐low attachment dish for 8 d to form EBs‐like spheres. The morphology of EBs was observed with a microscope then. In addition, total RNA was isolated to investigate gene expression of the three germ layers. Furthermore, the formed EBs were attached in gelatin precoated confocal dishes and underwent a differentiation process for 4 d. Immunofluorescence staining assay was performed to detect NESTIN, *α*‐SMA, and AFP expression.

##### Teratoma Formation

20 µg mL^−1^ MgAl‐LDH or 20 µg mL^−1^ MgFe‐LDH or LIF treated mESCs were collected in passages 3 and cells with a concentration of 1 × 10^7^ mL^−1^ in DMEM were resuspended with the same volume of Matrigel (Corning) on ice. 100 µL mixture were then subcutaneously injected into the flank of immunocompromised nude mice. 4 weeks after injection, teratomas from euthanized mice were obtained. Immunocompromised nude mice (6–8 weeks old, female) were purchased from the Shanghai Laboratory Animal Co. Ltd. (Shanghai, China) and housed in the Laboratory Animal Centre of Tongji Hospital of Tongji University. All animal experimental protocols were approved by the Institutional Research Ethics Committee of Tongji Hospital of Tongji University.

##### HE Staining

Teratomas were fixed in PFA, embedded in paraffin, and sectioned step by step. Paraffin‐embedded sections were deparaffinized in Histo‐Clear, rehydrated in ethanol, and rinsed with running water. The sections were then stained with hematoxylin solution for 3–5 min, followed by differentiation liquid and blue returning liquid treatment. Eosin staining was performed for 5 min after the sections were dehydrated. Finally, sections were dehydrated again and sealed with neutral balsam for histological analysis.

##### Immunohistochemistry

Immunohistochemistry for GFAP, *α*‐SMA, and AFP were conducted then. The paraffin sections were deparaffinized and rehydrated at first. For better exposure of target antigens, citric acid antigen retrieval buffer was applied. The sections were then placed in 3% hydrogen peroxide to block endogenous peroxidase activity followed by 3% BSA blocking. The primary antibodies were incubated overnight at 4 °C, and HRP labeled secondary antibody together with diaminobenzine (DAB) was used to visualize the positive staining. The nuclei were counterstained with hematoxylin solution subsequently.

##### mRNA Library Preparation for RNA‐Seq

mESCs were cultured in a) LIF, b) free of LIF, c) 20 µg mL^−1^ MgAl‐LDH, and d) 20 µg mL^−1^ MgFe‐LDH systems for 3 d. Each sample was defined as a1, a2, a3, b1, b2, b3, c1, c2, c3, d1, d2, and d3, respectively. Total RNA was isolated and RNA sequencing libraries were generated. Furthermore, the sequencing was performed on a BGISEQ‐500 system at The Beijing Genomics Institute (BGI) Company (Shenzhen, China).

##### Protein Extraction for DIA

mESCs were washed with cold PBS and collected by a cell scraper. a) LIF, b) free of LIF, c) 20 µg mL^−1^ MgAl‐LDH, and d) 20 µg mL^−1^ MgFe‐LDH were included and each sample was defined as a4, a5, a6, b4, b5, b6, c4, c5, c6, d4, d5, and d6, respectively. Cell pellets were resuspended in cytoplasmic protein extraction buffer (including 2 × 10^−3^ m EDTA and 1 × 10^−3^ m PMSF) for 5 min with gentle agitation, then 10 × 10^−3^ m Dithiothreitol (DTT) was added. A tissue grinder was applied to oscillate the samples and the samples were centrifuged at 25 000 g for 15 min to obtain the supernatant followed by incubated with 10 × 10^−3^ m DTT. The sample was boiled at 56 °C for 1 h, and a final concentration of 55 × 10^−3^ m iodacetamide (IAM) was added for 1 h incubation. Mix the sample with cold acetone and put them at −20 °C for 3 h. Repeat the above process until the supernatant is colorless. Centrifuge at 25 000 g for 15 min to obtain the sediment, and the precipitated proteins were lysed then. After centrifugation, the supernatant was carefully taken out and subjected to data independent acquisition (DIA) labeling followed by liquid chromatograph‐mass spectrometer (LC‐MS)/mass spectrometer (MS) and proteomic analysis (BGI Company, Shenzhen, China).

##### Dot Blot Analysis

A TIANamp Genomic DNA Kit (TIANGEN, Beijing) was applied for isolation of genomic DNA, and RNase A digestion was included. Subsequently, DNA samples were spotted on a dried nylon membrane pretreated with methanol. The membrane was set under ultraviolet for 30 min to crosslink the DNA. The crossed membrane was then washed with TBST, followed by blocking in 5% milk. Subsequently, the 5mc and 5hmc antibodies diluted in 5% nonfat milk were applied to the membrane at 4 °C overnight. The membrane was washed three times with TBST, then immersed with a secondary antibody. The immunoblots were then washed for 10 min three times in TBST and visualized by chemiluminescence with a Tanon scanner.

##### Quantification of Fe^2+^

Iron Assay Kit (Solarbio, Beijing) was applied for quantification of Fe^2+^ following the manufacturer's instructions. Briefly, cells were incubated with lysis buffer to release Fe^2+^. The supernatant was collected by centrifuging, incubated with working buffer at 95 °C for 5 min, and frozen by cold water then. 200 µL supernatant was obtained by incubating with 60 µL chloroform and centrifuging. The output was measured immediately on a microplate reader (optical density (OD) 520 nm).

##### Colocalization of Nanoparticles and LIFR

4 mg mL^−1^ MgFe‐LDH or MgAl‐LDH was incubated with 2 mg mL^−1^ FITC overnight at 4 °C to label LDH nanoparticles. mESCs treated with LDH‐FITC were then fixed by 4% PFA for 10 min. Cells were blocked with blocking buffer for 1 h and incubated with LIFR antibody for 2 h followed by secondary antibody for 2 h at room temperature. Confocal laser scanning microscope was applied to observe the colocalization of nanoparticles and LIFR then.

##### Statistical Analysis

All statistical data are shown of at least three independent experiments. For each analysis, the data were presented as the mean ± standard deviation or as values directly. All statistical analyses were performed using GraphPad Prism 8 software (GraphPad Software, San Diego, CA). Statistical significance was analyzed using one‐way analysis of variance unpaired or two‐tailed Student's *t*‐test. *p < 0.05 was regarded as significant.

## Conflict of Interest

The authors declare no conflict of interest.

## Supporting information

Supporting Information

## Data Availability

Research data are not shared.
