# Supplementary material for: MgFe‐LDH Nanoparticles: A Promising Leukemia Inhibitory Factor Replacement for Self‐Renewal and Pluripotency Maintenance in Cultured Mouse Embryonic Stem Cells
Source: Adv Sci (Weinh). 2021 Feb 25;8(9):2003535. doi: 10.1002/advs.202003535 (PMC8097378; doi:10.1002/advs.202003535)
Supplement: Supplementary file 1 — Supporting Information [file ADVS-8-2003535-s001.pdf]

© 2021 Wiley-VCH GmbH

ADVANCED SCIENCE
Open Access

Supporting Information

for *Adv. Sci.*, DOI: 10.1002/advs.202003535

MgFe-LDH nanoparticles: a promising leukemia inhibitory factor replacement for self-renewal and pluripotency maintenance in cultured mouse embryonic stem cells

*Xiaolie He, Yanjing Zhu, Li Yang, Zhaojie Wang, Zekun Wang, Jianhao Feng, Xuejun Wen, Liming Cheng, and Rongrong Zhu**

Supporting Information

MgFe-LDH nanoparticles: a promising leukemia inhibitory factor replacement for self-renewal and pluripotency maintenance in cultured mouse embryonic stem cells

*Xiaolie He, Yanjing Zhu, Li Yang, Zhaojie Wang, Zekun Wang, Jianhao Feng, Xuejun Wen, Liming Cheng, and Rongrong Zhu**

Dr. X. He, Dr. Y. Zhu, Dr. L. Yang, Dr. Z. Wang, Z. Wang, J. Feng, Prof. X. Wen, Prof. L. Cheng, Prof. R. Zhu

Key Laboratory of Spine and Spinal Cord Injury Repair and Regeneration of Ministry of Education, Orthopaedic Department of Tongji Hospital, School of Life Science and Technology, Tongji University, 389 Xincun Road, 200065, Shanghai, P. R. China

Prof. X. Wen

Department of Chemical and Life Science Engineering, School of Engineering, Virginia Commonwealth University, Richmond, VA 23284, USA

E-mail: rrzhu@tongji.edu.cn

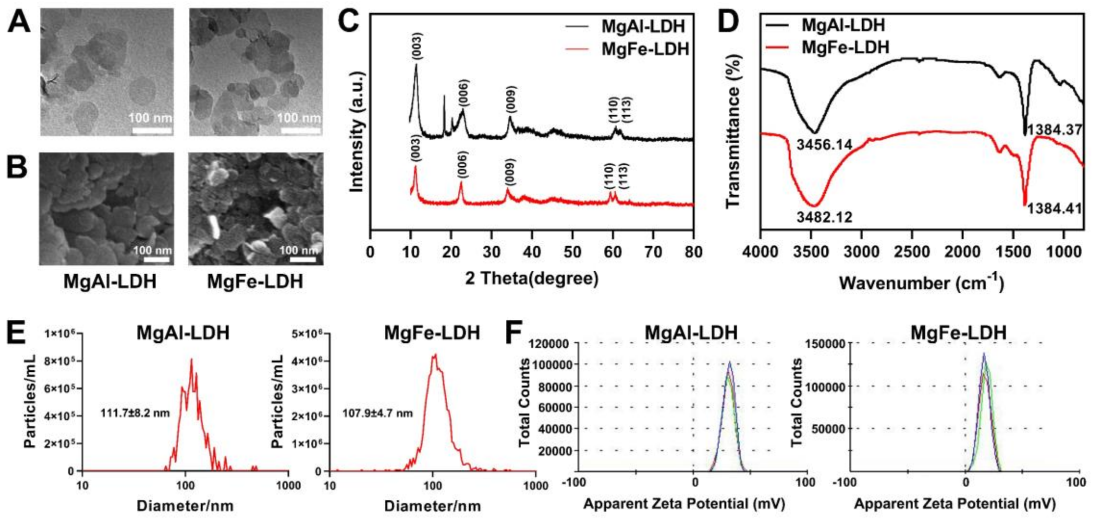

Figure S1. Synthesis and characterization of MgFe-LDH and MgAl-LDH nanoparticles. (A) TEM images of MgAl-LDH (left) and MgFe-LDH (right). (B) SEM images of MgAl-LDH (left) and MgFe-LDH (right). (C) X-ray diffraction pattern analysis. (D) FTIR spectroscopy. (E) Particle size distribution of nanoparticles. (F) Mean Zeta potential of nanoparticles measured by Nano Zetasizer.

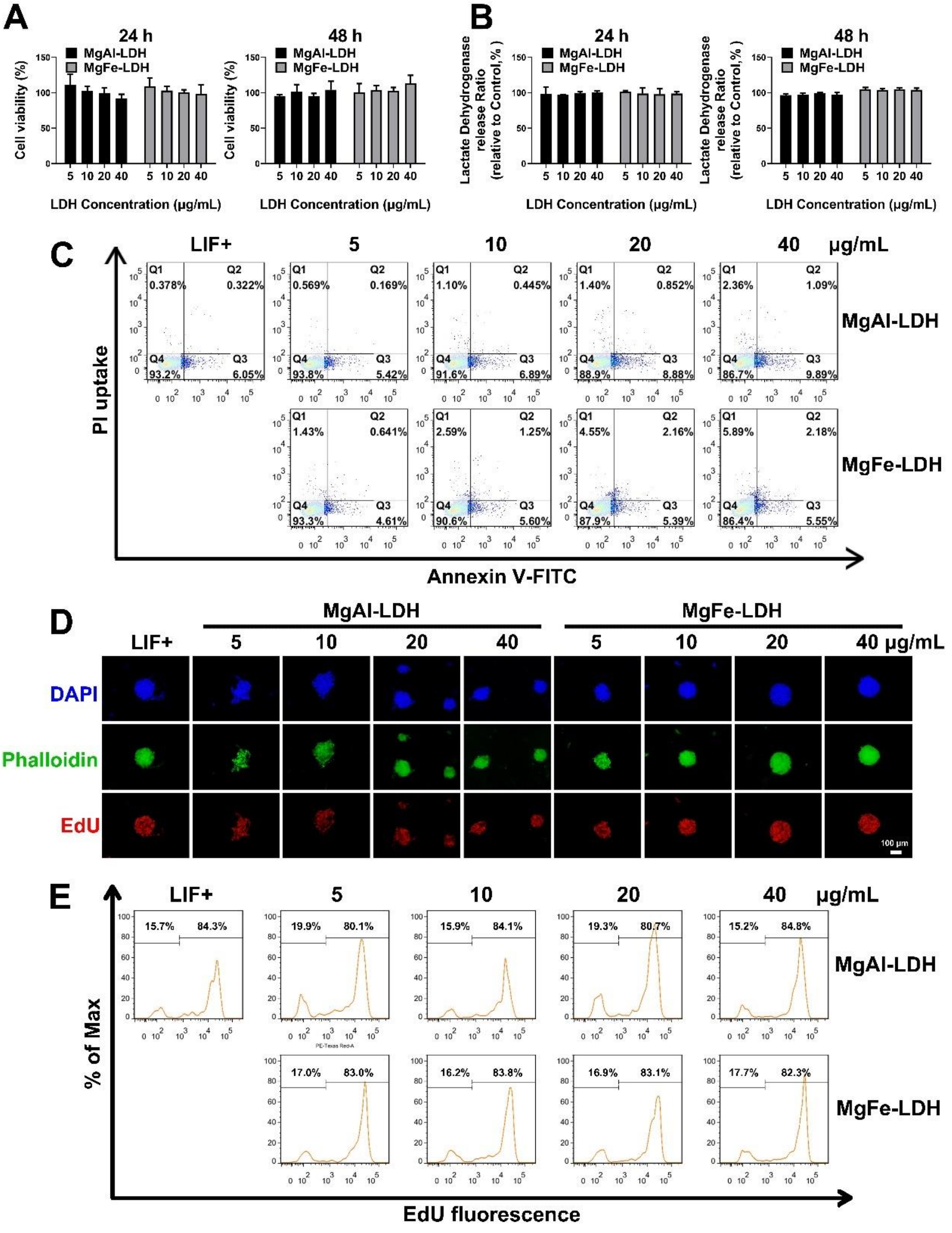

Figure S2. Comparison of MgFe-LDH and MgAl-LDH biocompatibility with mESCs. (A) Cell survival analysis following treatment with 5, 10, 20, and 40 μg/mL of MgAl-LDH and MgFe-LDH, detected by CCK-8 Kit. (B) Detection of lactic dehydrogenase release from mESCs treated by nanoparticles reflected the integrity of their cell membrane treated by nanoparticles. (C) Flow cytometry analysis of mESC apoptosis by Annexin V-FITC/PI staining. (D) The proliferation rate of mESCs treated with nanoparticles evaluated by EdU proliferation assay. (E) Quantification of proliferating cells by FACS analysis.

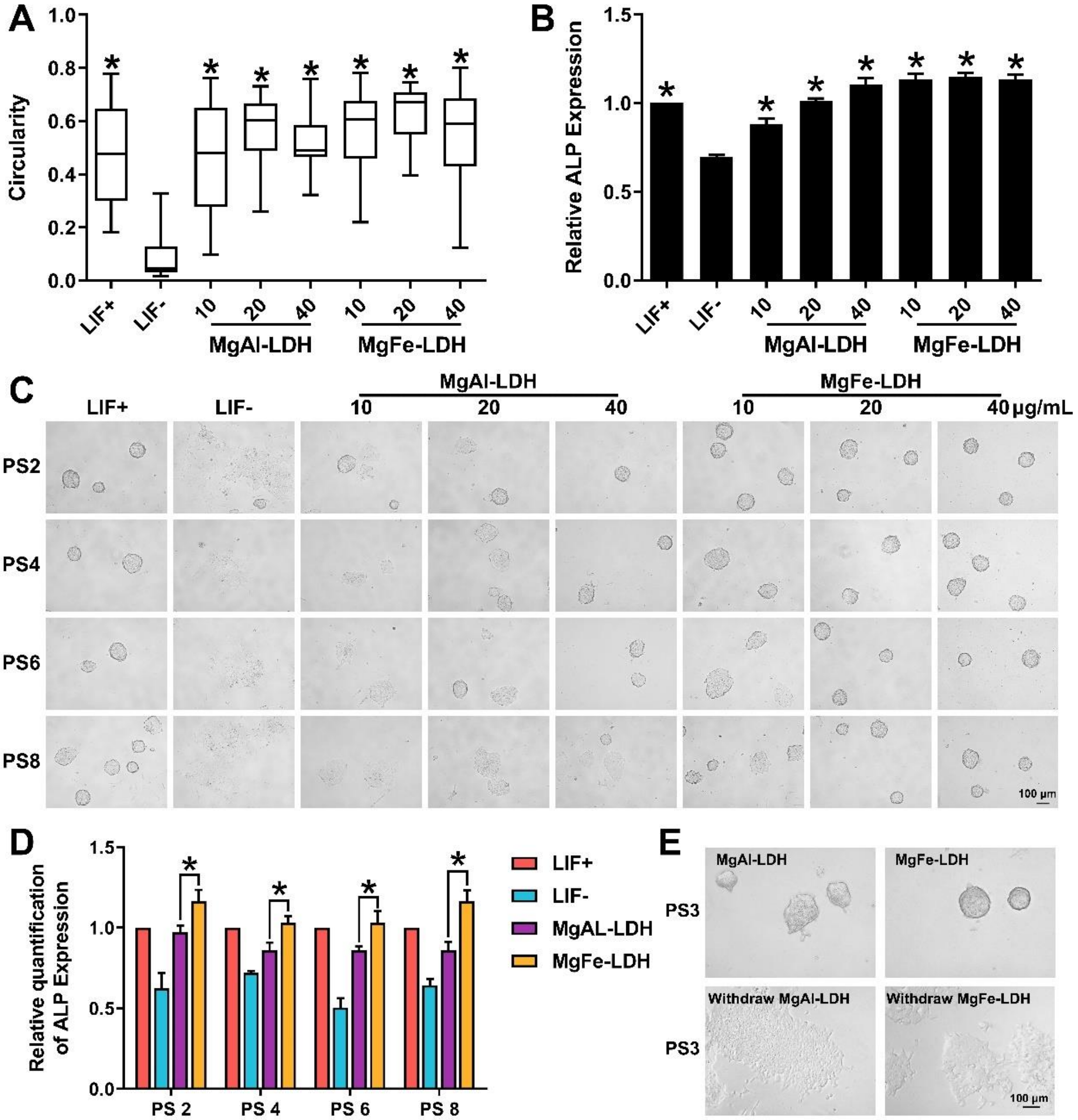

Figure S3. Comparison of MgFe-LDH and MgAl-LDH on their ability to maintain mESC self-renewal. (A) Quantification of colony circularity under various conditions. (B) Relative ALP expression for different treatments. (C) Bright-field images of mESCs at indicated passage: 2, 4, 6, and 8. (D) Relative quantification of ALP expression at different passages. * represents $p < 0.05$, when compared to the MgAl-LDH group. (E) Withdrawal of nanoparticles in PS3 resulted in the differentiation of the mESCs.

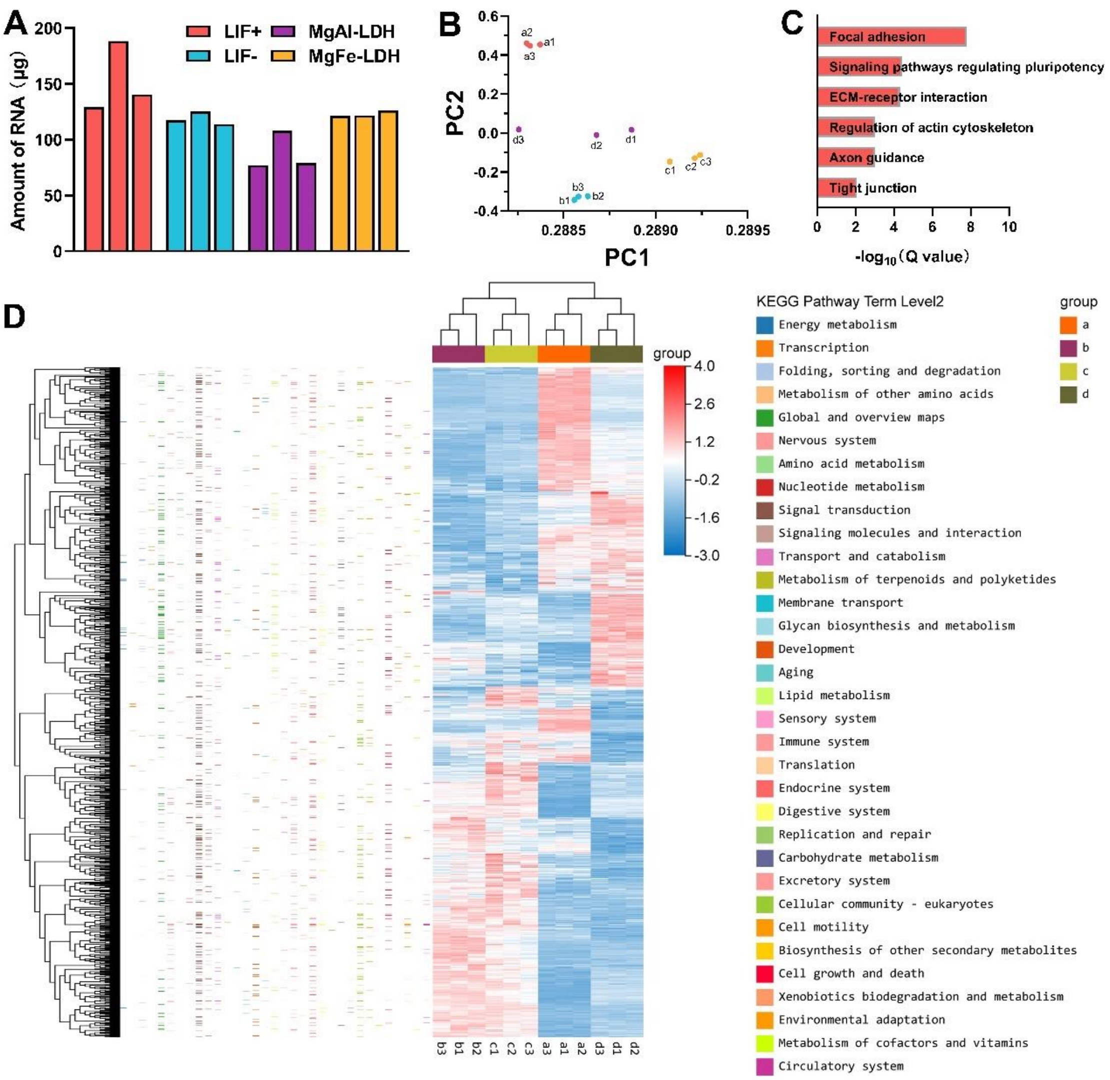

Figure S4. Transcriptomic analysis to compare mRNA expression profiles between the MgFe-LDH and MgAl-LDH groups. (A) RNA was isolated, and subsequently quantified by Nanodrop. (B) 2D PCA results for the LIF+ (a), LIF- (b), MgAl-LDH (c), and MgFe-LDH (d) groups using DEGs of a-VS-b. (C) KEGG pathway analysis of the DEGs between the MgAl-LDH (c) and MgFe-LDH (d) groups. (D) Clustering analysis of DEGs between the MgFe-LDH and MgAl-LDH groups.

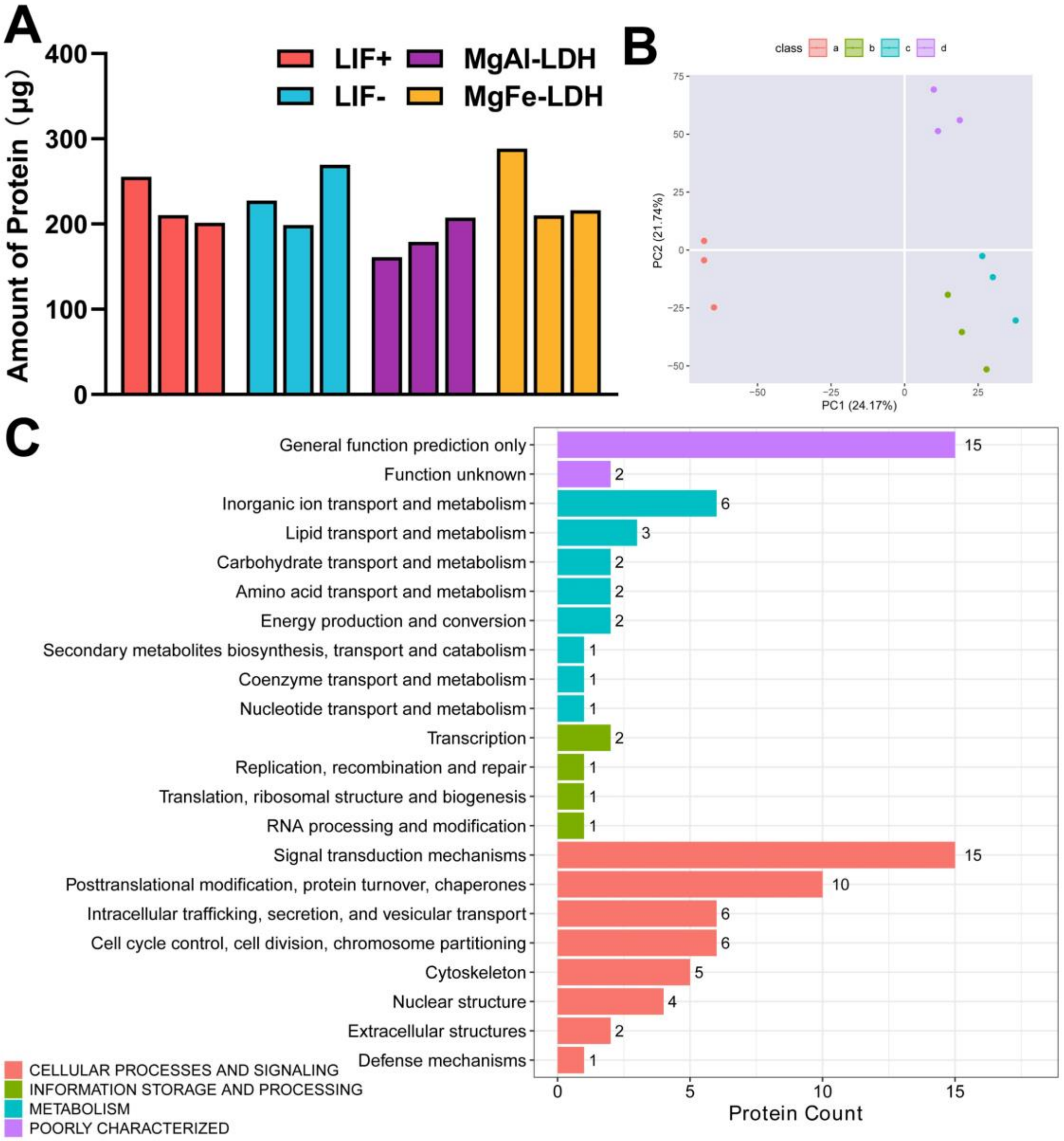

Figure S5. Proteomic analysis to compare protein expression profiles between the MgFe-LDH and MgAl-LDH groups. (A) Extracted protein was quantified. (B) 2D PCA analysis of the LIF+ (a), LIF- (b), MgAl-LDH (c), and MgFe-LDH (d) groups. (C) KOG pathway analysis of the DEPs between the MgAl-LDH and MgFe-LDH groups.

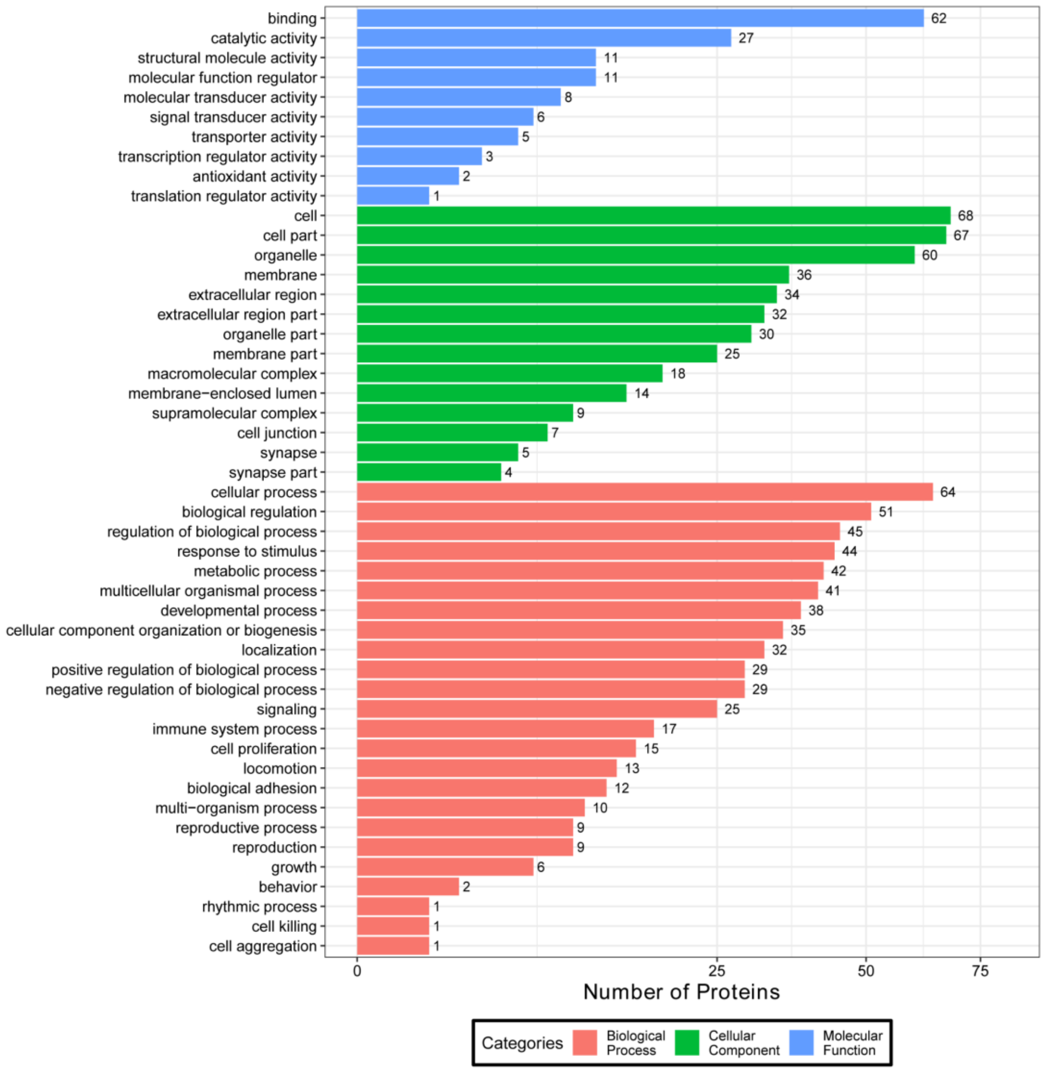

Figure S6. Gene ontology enrichment analysis of the DEPs between the MgFe-LDH and MgAl-LDH groups.

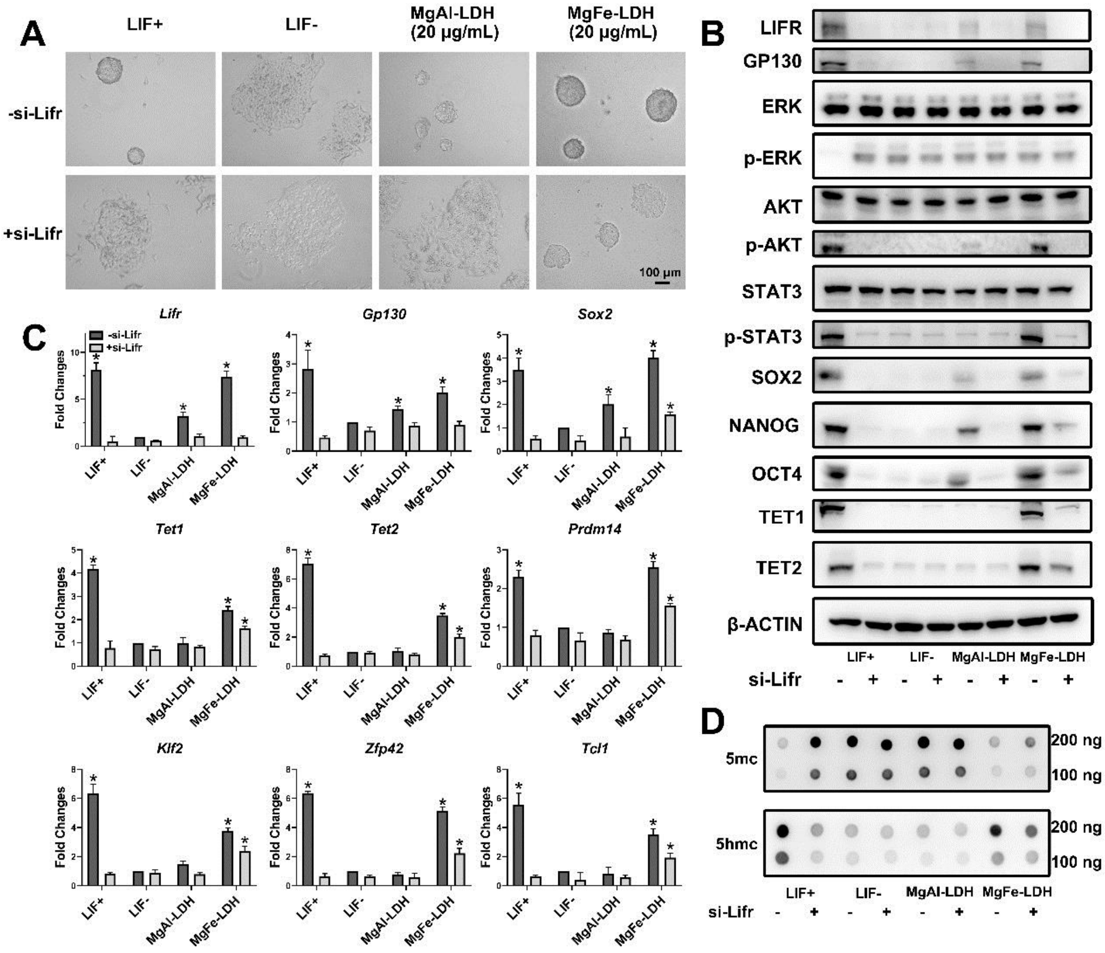

Figure S7. LIFR knockdown by using si-Lifr. (A) Bright-field images of mESCs cultured in media containing si-Lifr, under different treatment conditions. (B) Protein expression changes for mESCs, with or without si-Lifr, subjected to the requisite treatment. (C) mRNA expression changes following the si-Lifr treatment. * represents $p < 0.05$, when compared to the LIF- group. (D) Global 5mc and 5hmc levels detected by dot blot.

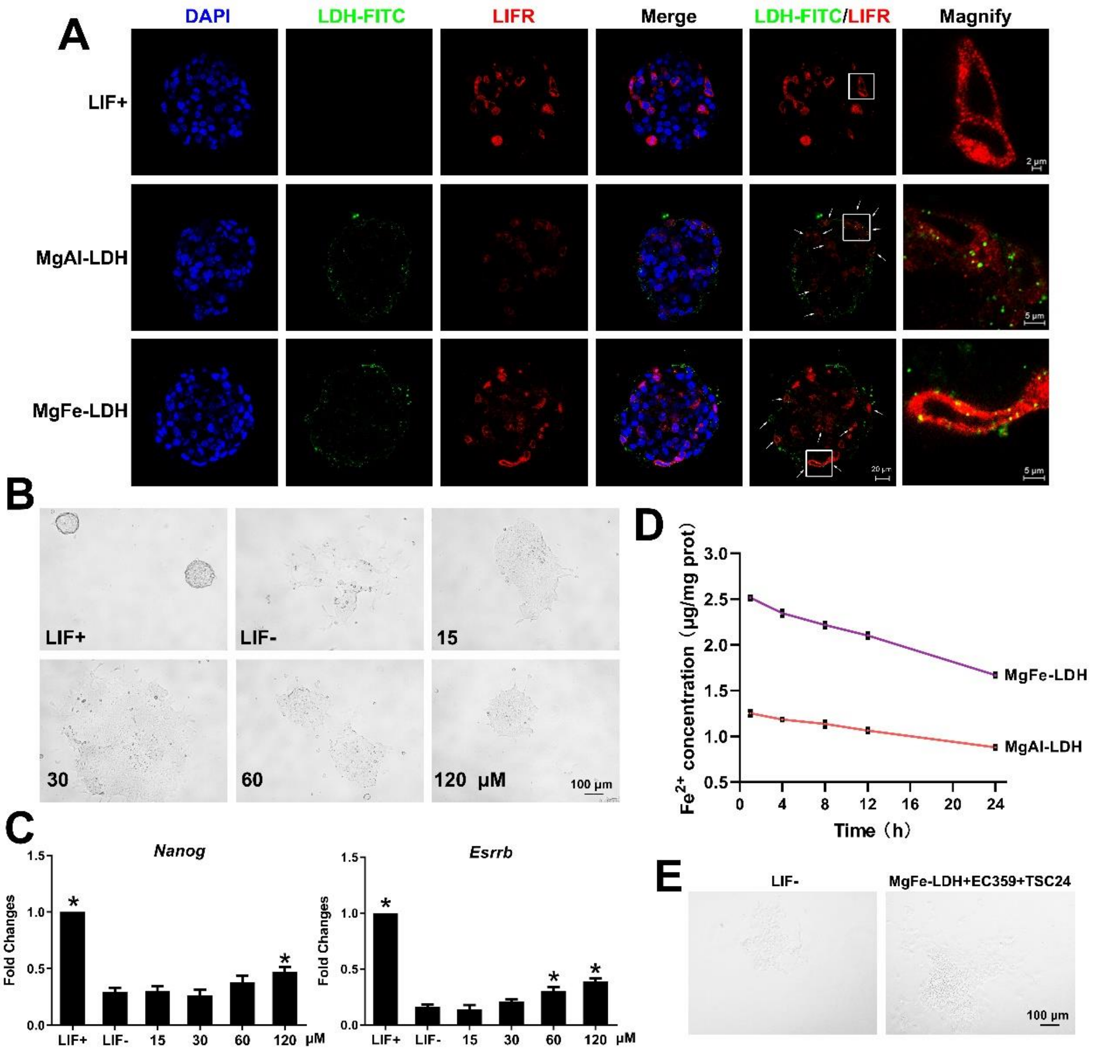

Figure S8. (A) Colocalization of nanoparticles and LIFR were observed using confocal laser scanning microscope. (B) Function of ferric nitrate (used in MgFe-LDH fabrication) in supporting mESC self-renewal. (C) qPCR analysis of pluripotency genes (*Nanog* and *Esrrb*). * represents $p < 0.05$, when compared to the LIF- treatment. (D) The MgFe-LDH group provided additional Fe^{2+}, as compared to MgAl-LDH group in mESC culture. (E) Co-administration of MgFe-LDH, EC359, and TSC24 resulted in cell differentiation.

Table S1. The list of primer sequences.

Gene	Forward primer	Reverse primer
Gapdh	GTGTTCCTACCCCCAATGTGT	ATTGTCATACCAGGAAATGAGCTT
Nanog	TCTTCCTGGTCCCCACAGTTT	GCAAGAATAGTTCTCGGGATGAA
Esrrb	GCACCTGGGCTCTAGTTGC	TACAGTCCTCGTAGCTCTTGC
Rex-1	CGATGCTGGAGTGTCCTCAAG	GCCACACTCTGCACACACGT
Nestin	CCCTGAAGTCGAGGAGCTG	CTGCTGCACCTCTAAGCGA
Eomes	GCGCATGTTTCCTTTCTTGAG	GGTCGGCCAGAACCACTTC
Cxcr4	GAAGTGGGGTCTGGAGACTAT	TTGCCGACTATGCCAGTCAAG
Sox1	GCGGAGTGGAAACTTTTGTCC	CGGGAAGCGTGTACTTATCCTT
Kdr	TTTGGCAAATACAACCCTTCAGA	GCAGAAGATACTGTCACCACC
α-SMA	CCCAACTGGGACCACATGG	TACATGCGGGGGACATTGAAG
Gata4	CCCTACCCAGCCTACATGG	ACATATCGAGATTGGGGTGTCT
Gata6	TTGCTCCGGTAACAGCAGTG	GTGGTCGCTTGTGTAGAAGGA
Lifr	TACGTCGGCAGACTCGATATT	TGGGCGTATCTCTCTCTCCTT

Gp130	CCGTGTGGTTACATCTACCCT	CGTGGTTCTGTTGATGACAGTG
Sox2	CGATGCTGGAGTGTCCTCAAG	GCCACACTCTGCACACACGT
Tet1	CCATTCTCACAAGGACATTCACA	GCAGGACGTGGAGTTGTTCA
Tet2	GCCATTCTCAGGAGTCACTGC	ACTTCTCGATTGTCTTCTCTATTGAGG
Prdm14	CTCTTGATGCTTTTCGGATGACT	GTGACAATTTGTACCAGGGCA
Klf2	CGCCGCCACACATACTTG	AACTTCCAGCCGCATCCTT
Zfp42	CGATGCTGGAGTGTCCTCAAG	GCCACACTCTGCACACACGT
Tcl1	CTCCATGTATTGGCAGATCCTGTA	CTCCGAGTCTATCAGTTCAAGCAA
